# PARG regulates the proteasomal degradation of TARG1

**DOI:** 10.1016/j.celrep.2025.116789

**Published:** 2026-01-06

**Authors:** Joséphine Groslambert, Sara C. Buch-Larsen, Ivo A. Hendriks, Robert Kurzbauer, Jonas D. Elsborg, Chatrin Chatrin, Thomas Agnew, Callum Henfrey, Michael Tellier, Evgeniia Prokhorova, Song My Hoang, Jonathan Barosso-Gonzalez, Roderick J. O’Sullivan, Tim Clausen, Michael L. Nielsen, Ivan Ahel

**Affiliations:** 1 Sir William Dunn School of Pathology, University of Oxford, Oxford, UK; 2 Novo Nordisk Foundation Center for Protein Research, Department of Cellular and Molecular Medicine, Faculty of Health and Medical Sciences, University of Copenhagen, Copenhagen, Denmark; 3 Research Institute of Molecular Pathology, Vienna BioCenter (VBC), Vienna, Austria; 4 Department of Molecular and Cell Biology, University of Leicester, Leicester, UK; 5 Department of Pharmacology and Chemical Biology, UPMC Hillman Cancer, University of Pittsburgh, Pittsburgh, PA, USA; 6 Lead contact

## Abstract

ADP-ribosylation (ADPr) is a reversible modification of macromolecules critical for the regulation of genome stability, stress responses, and proteostasis. While the roles of ADPr transferases such as PARP1/2 and TNKS1/2 are well established, the functions and regulatory mechanisms of ADPr hydrolases are still poorly understood. Here, we identify a function of the poly(ADP-ribose) glycohydrolase PARG in regulating protein degradation. Using quantitative proteomics, we show that PARG inhibition depletes protein levels of the mono-ADPr hydrolase TARG1. We demonstrate that this TARG1 depletion is both PAR and proteasome dependent and identify the E3 ubiquitin ligases HUWE1 and TRIP12 as mediators of this process. Our findings establish TARG1 as a substrate of PAR-dependent protein degradation and uncover a PARG-dependent mechanism controlling its stability. This work highlights an interplay between the two ADP-ribosyl hydrolases, with implications for the refinement of PARG-targeted therapeutic strategies.

## In brief

Groslambert et al. reveal that PARG controls the PAR-dependent proteasomal degradation of TARG1. The authors additionally identify the E3 ligases HUWE1 and TRIP12 as key factors that target TARG1 for degradation, further establishing their roles as PAR-dependent E3 ligases.

## Graphical Abstract

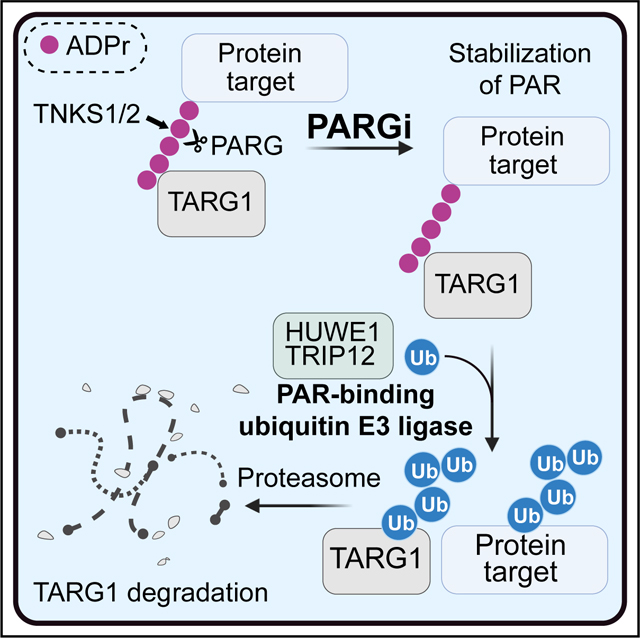

## INTRODUCTION

ADP-ribosylation (ADPr) is a reversible modification of macromolecules involved in regulating diverse cellular processes, including DNA repair, transcription, stress responses, and protein degradation.^1^ This modification involves the transfer of ADP-ribose units from NAD^+^ to target proteins and exists in two principal forms: mono-ADP-ribosylation (MAR) and poly-ADP-ribosylation (PAR).^2^ While MAR involves the addition of a single ADP-ribose unit, PAR consists of branched or linear chains of multiple ADP-ribose moieties.^3^

PAR synthesis is catalyzed by a limited subset of ADP-ribosyltransferases—specifically, PARP1, PARP2, and the tankyrases TNKS1 and TNKS2—distinguishing these enzymes from the broader family of MARylating PARPs.^1,4,5^ The best-characterized role of PARP1/2 lies in regulating the DNA damage response (DDR), where it mediates chromatin remodeling and recruits DNA repair factors to the sites of damage.^6,7^ In contrast, TNKS1/2 is best known for its role in regulating protein degradation through PAR-dependent ubiquitylation (PARdU)—a process in which TNKS1/2-catalyzed PARylation of substrates acts as an activating signal for specific E3 ligases, thereby targeting them for ubiquitylation and subsequent degradation via the proteasome.^8,9^ RNF146, the best characterized PAR-dependent E3 ligase, contains an iso-ADPr binding WWE domain that, upon interaction with PAR chains synthesized by TNKS1/2, activates its E3 ubiquitin ligase activity.^10,11^ Through PARdU, the coordinated activities of TNKS1/2 and RNF146 regulate the stability of key factors, such as Axin, 3BP2, PTEN, and AMOT, thereby modulating essential signaling pathways, including Wnt signaling.^12–16^ Moreover, TRIP12, another WWE domain-containing E3 ligase, has been shown to promote the ubiquitylation and degradation of PARP1 in a PAR-dependent manner, thereby limiting PARP1 trapping and resulting in replication stress, underscoring the physiological relevance of PARdU regulation.^17^

The removal of ADP-ribose modifications is mediated by dedicated hydrolases, ensuring precise temporal and spatial control over ADPr signaling. A central component of this process is PARG, the primary enzyme responsible for degrading PAR chains.^18–20^ The efficient removal of PARylation occurs via a two-step mechanism: PARG first cleaves the glycosidic bonds within the PAR chains, and specialized MAR hydrolases then remove the terminal ADP-ribose unit covalently attached to a protein.^21,22^ This process involves a diverse set of MAR hydrolases with distinct substrate preferences: ARH3 primarily targets serine-linked MAR,^21,23^ both ARH3 and PARG contribute to tyrosine-linked MAR removal,^24^ and glutamate- or aspartate-linked MAR can be reversed by TARG1, MACROD1, MACROD2, PARP9, and PARP14.^25–28^ Notably, there is functional redundancy and interplay among these hydrolases,^25,29^ underscoring the complexity of ADPr signaling. For instance, we and others have previously shown that disruption of MAR reversal can sensitize cells to PARG inhibition. Specifically, ARH3 and TARG1 have emerged as synthetic lethal interactors of PARG, indicating that these MAR hydrolases functionally act in concert with PARG to prevent the toxic accumulation of ADP-ribose modifications.^21,25,30^ The combined hydrolytic activity of TARG1 and PARG plays a critical role in maintaining genomic stability, in part by suppressing replication stress,^25^ although the underlying molecular mechanisms remain to be fully defined.

Here, we further investigate the interplay between PARG and TARG1 and demonstrate that PARG inhibition leads to a marked reduction in TARG1 protein levels. Using mass spectrometry-based quantitative proteomics, we show that PARG inhibition results in the depletion of TARG1, along with several other proteins. Further elucidation of the mechanisms underlying this deregulation reveals that TARG1 depletion upon PARG inhibition is both proteasome and PAR dependent. Moreover, we identify the E3 ubiquitin ligases HUWE1 and TRIP12 as key mediators of this process. Our findings thus establish TARG1 as a substrate of PAR-dependent protein degradation. Collectively, our study reveals a mechanism by which PARG safe-guards TARG1 protein stability, highlighting an additional layer of functional interplay between these two enzymes.

## RESULTS

### PARG inhibition leads to TARG1 protein depletion

**Figure 1.**
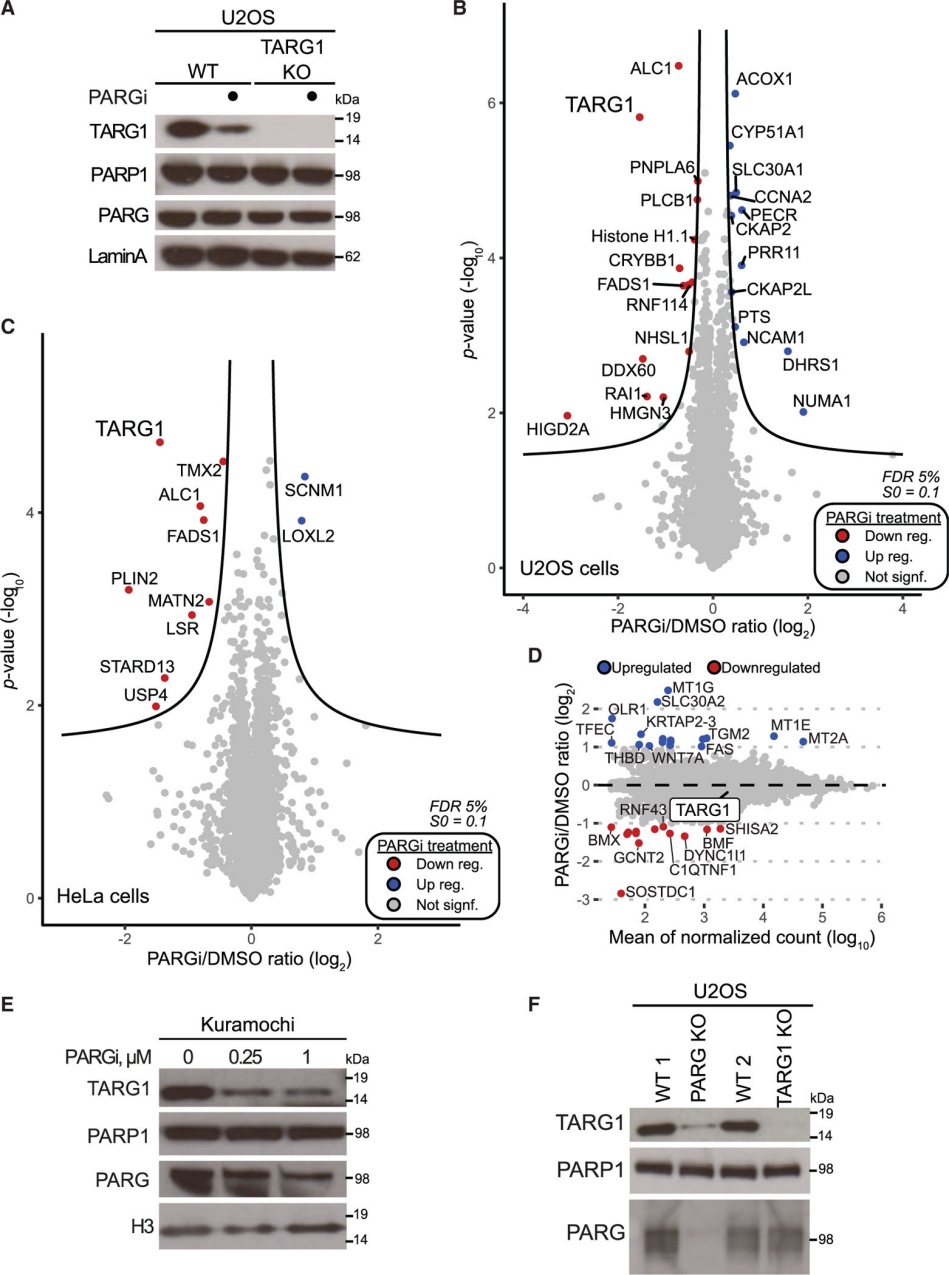
TARG1 protein levels are depleted upon loss of PARG catalytic activity (A) U2OS cells were treated with DMSO or 10 μM PARGi for 2 days. TARG1 levels were analyzed by western blotting. (B and C) U2OS (B) and HeLa (C) cells were treated with DMSO or 25 μM PARGi for 4 days prior to LC-MS analysis. The volcano plot shows log_2_-fold change of proteins plotted against the corresponding *p* value. Significance was determined by Student’s *t* test, with a false discovery rate threshold of 5% and fudge factor S0 = 0.1; *n* = 4. (D) MA plot showing differentially expressed genes in WT U2OS cells treated with 25 μM PARGi for 4 days against the DMSO control. *n* = 3, adjusted *p* < 0.05, absolute log_2_fold change >1.0. (E) Kuramochi WT cells were treated with DMSO, 0.25 μM and 1 μM PARGi for 4 days. TARG1 levels were analyzed by western blotting. (F) U2OS WT 1 and WT 2 are the respective clonal controls for U2OS PARG KO and TARG1 KO cells. TARG1 levels were analyzed by western blotting. See also Figure S1 and Data S1.

We previously established U2OS control and TARG1-knockout (KO) cell lines to examine the interplay between PARG and TARG1.^25^ Using these cells, we noted that treatment with the PARG inhibitor (PARGi) PDD00017273^31^ led to reduced TARG1 levels in U2OS cells (Figure 1A). To examine this effect in more detail, we performed a time-course experiment and found that the accumulation of ADPr induced by PARG inhibition peaked after 24 h of treatment and remained elevated throughout the 96 h treatment (Figure S1A). A decrease in TARG1 levels was already evident after 24 h of PARGi treatment and became progressively more pronounced with longer treatment (Figure S1A), indicating that sustained, unresolved PAR accumulation correlates with a gradual depletion of TARG1 over time.

To characterise this effect on a global scale, we next assessed the proteomic impact of PARG inhibition using mass spectrometry-based proteomics. To this end, we cultured U2OS and HeLa cells in the presence of PARGi or DMSO (mock control) for 4 days to capture proteome-wide changes arising upon sustained PARG inhibition, and analyzed their total proteomes using label-free quantitative liquid chromatography-mass spectrometry (LC-MS) analysis (Figures 1B and 1C). Across all cell lines and conditions, we identified 9,955 protein groups. After stringent filtering, 7,220 and 7,576 proteins could be fully quantified via at least 3 precursors for HeLa and U2OS cells, respectively.

Proteomes had excellent reproducibility within sample groups, with median coefficient variations of ~5%–7% (Figure S1B). In U2OS cells, 26 proteins were significantly altered upon PARGi treatment, with 12 proteins showing increased and 14 proteins showing decreased abundance (Figure 1B). Furthermore, in HeLa cells, we found 12 proteins to be significantly affected, with 3 increased and 9 proteins decreased (Figure 1C). These data reveal that PARG inhibition perturbs the stability of a small subset of proteins, and provide a valuable resource for further investigation of its molecular and cellular effects. Of note, the PAR-dependent chromatin remodeler ALC1^32^ was found to be reduced in both cell lines upon PARGi treatment (Figures 1 B and 1C; Data S1). Importantly, TARG1 emerged as one of the most strongly depleted proteins in both cell lines, suggesting a conserved response (Figures 1B and 1C; Data S1), which prompted us to investigate the underlying mechanism of this regulation.

We wondered if the observed reduction of TARG1 was due to transcriptional changes, and addressed this by performing RNA sequencing (RNA-seq) on U2OS wild-type (WT) cells with the treatments, as previously described. No significant changes in TARG1 mRNA expression were detected, suggesting that TARG1 stability is regulated post-transcriptionally (Figures 1D and Data S1). While we observed a moderate overall correlation between the proteome and transcriptome (Pearson’s r = 0.49; Figure S1C), the PARGi-regulated proteome correlated poorly with the PARGi-regulated transcriptome (Pearson’s r = 0.15; Figure S1D). In fact, none of the significantly regulated proteins from either cell line identified by proteomics overlapped with the RNA-seq hits (Figures 1B and 1D; Data S1). Together, these results support the idea that PARGi-dependent regulation of protein levels is more likely to occur through post-transcriptional mechanisms.

While we previously demonstrated that PARG and TARG1 cooperate to suppress genomic instability by preventing the accumulation of PARylation,^25^ the robust suppression of TARG1 protein levels following PARG inhibition revealed here suggests an additional layer of functional interplay between these enzymes and uncovers a previously unappreciated role for PARG in regulating protein levels.

Next, we investigated whether the depleting effect of PARG inhibition on TARG1 also occurs in the clinically relevant Kuramochi ovarian cancer cell line, which is known to be sensitive to PARGi.^33–35^ Treatment with PARGi at concentrations as low as 0.25 μM resulted in a striking reduction of TARG1 levels (Figure 1E). These findings further support that the effect of PARG inhibition on TARG1 levels is conserved across a diverse range of cell types. Finally, to verify that the reduction in TARG1 levels was not the result of off-target effects of the PARG inhibitor used, we examined TARG1 levels in U2OS PARG-KO cells. Akin to PARG inhibition, PARG loss phenocopied TARG1 depletion as shown by the pronounced reduction in TARG1 levels in U2OS PARG-KO cells compared to the clonal control (Figure 1F). This strongly suggests that the observed reduction in TARG1 levels upon PARG inhibition is caused by the loss of PARG catalytic activity as opposed to any putative off-target effects.

### The PARGi-induced depletion of TARG1 is PARylation and proteasome dependent

**Figure 2.**
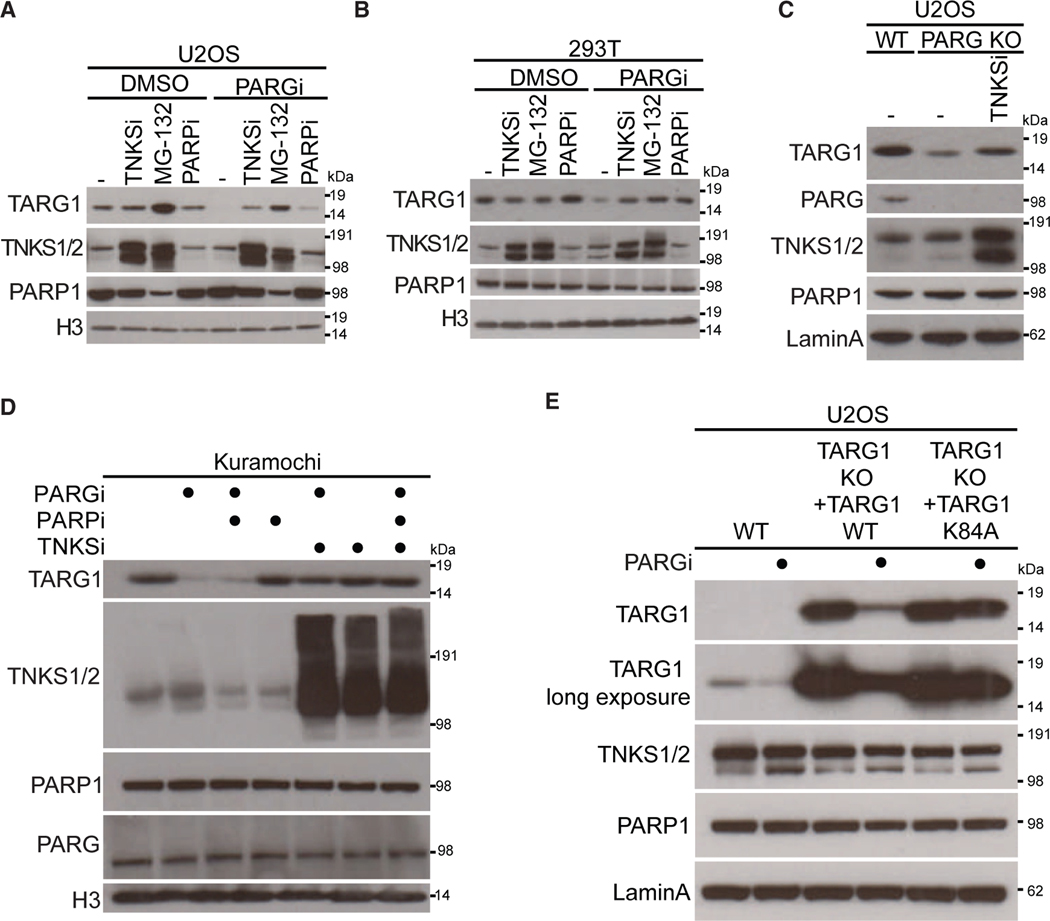
PARG inhibition leads to the proteasomal degradation of TARG1 in a PARylation-dependent manner (A and B) U2OS (A) and 293T (B) cells were treated with DMSO, 10 μM PARGi, 10 μM PARGi and 1 μM TNKSi, or 10 μM PARGi and 1 μM PARPi for 3 days. 2.5 μM MG-132 was added for the last 24 h of PARGi treatment. TARG1 and TNKS1/2 levels were analyzed by western blotting. The higher TNKS1/2 band corresponds to TNKS1 (142 kDa), while the lower band corresponds to TNKS2 (127 kDa). Three independent biological replicates were performed with similar results. (C) U2OS cells were treated with DMSO or 1 μM TNKSi for 2 days. TARG1 and TNKS1/2 levels were analyzed by western blotting. (D) Kuramochi cells were treated with DMSO, 1 μM TNKSi, or different combinations of 10 μM PARGi, 1 μM PARPi, and 1 μM TNKSi as indicated for 4 days. TARG1 and TNKS1/2 levels were analyzed by western blotting. (E) U2OS WT and TARG1-KO cells complemented with TARG1 WT or catalytically inactive K84A mutant were treated with 10 μM PARGi for 3 days. TARG1 levels were analyzed by western blotting. See also Figure S2.

Next, we examined whether the PARGi-induced depletion of TARG1 was dependent on the proteasome pathway, the primary protein degradation mechanism in eukaryotic cells. We treated U2OS cells with a proteasome inhibitor (MG-132) and found that it completely reversed PARGi-induced TARG1 depletion (Figures 2A and S2A). This finding demonstrates that PARGi treatment stimulates TARG1 degradation via the proteasome pathway. Notably, treatment with MG-132 also led to an increase in TARG1 protein levels in DMSO-treated U2OS cells (Figures 2A and S2A), showing that the degradation of TARG1 is regulated by the proteasome pathway under both basal and PARG-inhibited conditions. To validate the generalizability of this mechanism, we extended our investigation to additional cell lines and showed that 293T cells also displayed TARG1 depletion upon PARG inhibition (Figure 2B). In 293T cells, the rescuing effect of MG-132 on TARG1 levels further confirmed the proteasomal dependence of this protein degradation mechanism. Treatment of U2OS cells with MLN4924, an inhibitor of Cullin-RING ligases—the largest class of E3 ubiquitin ligases—did not rescue PARGi-induced TARG1 degradation, suggesting that TARG1 turnover is mediated by a different class of E3 ligases (Figure S2A).

The PARdU pathway connects ADPr signaling to protein degradation by using PAR chains as degradation signals that recruit and activate PAR-binding E3 ligases, which subsequently target proteins for proteasomal degradation.^8^ We thus hypothesized that TARG1 degradation could be regulated via a PARdU mechanism, with PARG, the primary PAR hydrolase, counteracting this degradation signal. This would explain the enhanced TARG1 degradation observed upon PARG inhibition (Figure 1A). To test this, we examined whether blocking PAR chains formation by inhibiting TNKS1/2, and PARP1/2 could rescue TARG1 degradation. In U2OS WT cells, addition of the tankyrase inhibitor (TNKSi) XAV939^12^ to PARG-inhibited cells nearly fully restored TARG1 levels to those of the DMSO-treated levels (Figure 2A). A similar rescuing effect was observed in U2OS PARG KO cells (Figure 2C) and in PARGi-treated 293T and Kuramochi cells (Figures 2B and 2D). These results indicate that TNKS1/2 activity drives TARG1 degradation upon PARG inhibition across these different cell types. Notably, TNKSi treatment also stabilized TNKS1/2 levels in all examined cell lines (Figures 2A–2D), confirming target engagement, as TNKS1/2 autoPARylation promotes its own proteasomal degradation.^11,36^ On the other hand, treatment with the PARP inhibitor (PARPi) olaparib had variable effects on TARG1 levels. In Kuramochi cells, it failed to counteract TARG1 degradation; in U2OS cells, it partially rescued TARG1 levels; and in 293T cells, it strongly restored TARG1 protein levels (Figures 2A, 2B, and 2D). Together, these findings support a model in which TARG1 turnover is regulated via a PARdU pathway, with PARylation by TNKS1/2 and/or PARP1/2 promoting its degradation. Our data suggest that PAR chains mediating TARG1 degradation are primarily synthesized by TNKS1/2 in U2OS and Kuramochi cells (Figures 2A, 2C, and 2D), whereas in 293T cells, both PARP1/2 and TNKS1/2 contribute to this process (Figure 2B). Although the involvement of PARP1/2 and TNKS1/2 in promoting TARG1 degradation appears to vary between cell lines, the role of PARG in counteracting this degradation is conserved.

TARG1 was previously shown to interact with PARP1 in a PAR-dependent manner, likely through binding to auto-PARylation.^26^ We thus sought to determine whether the PAR-binding ability of TARG1 is required for its degradation upon PARG inhibition. Using U2OS TARG1-KO cell lines stably overexpressing either WT TARG1 or the PAR-binding-deficient K84A mutant,^26^ we found that PARGi treatment led to pronounced depletion of WT TARG1, whereas levels of the K84A mutant remained unaffected (Figure 2E). These findings suggest that PAR binding is a necessary step for TARG1 degradation in response to PARG inhibition. Consistent with this, immunoprecipitation experiments showed that TARG1 WT, but not K84A mutant, interacts with TNKS1/2 and PARP1/2 (Figure S2B), suggesting the importance of PARylation in mediating these interactions. Notably, TARG1 did not bind to the MARylating PARP3 transferase, further supporting the requirement for PAR chains in mediating TARG1 complex formation. Although the functional significance of these interactions remains to be fully elucidated, they may reflect spatial proximity to PARylation events that facilitate TARG1 recruitment and subsequent degradation. Altogether, our results demonstrate that the degradation of TARG1 is PAR- and proteasome-dependent.

### The E3 ubiquitin ligases HUWE1 and TRIP12 target TARG1 for proteasomal degradation

**Figure 3.**
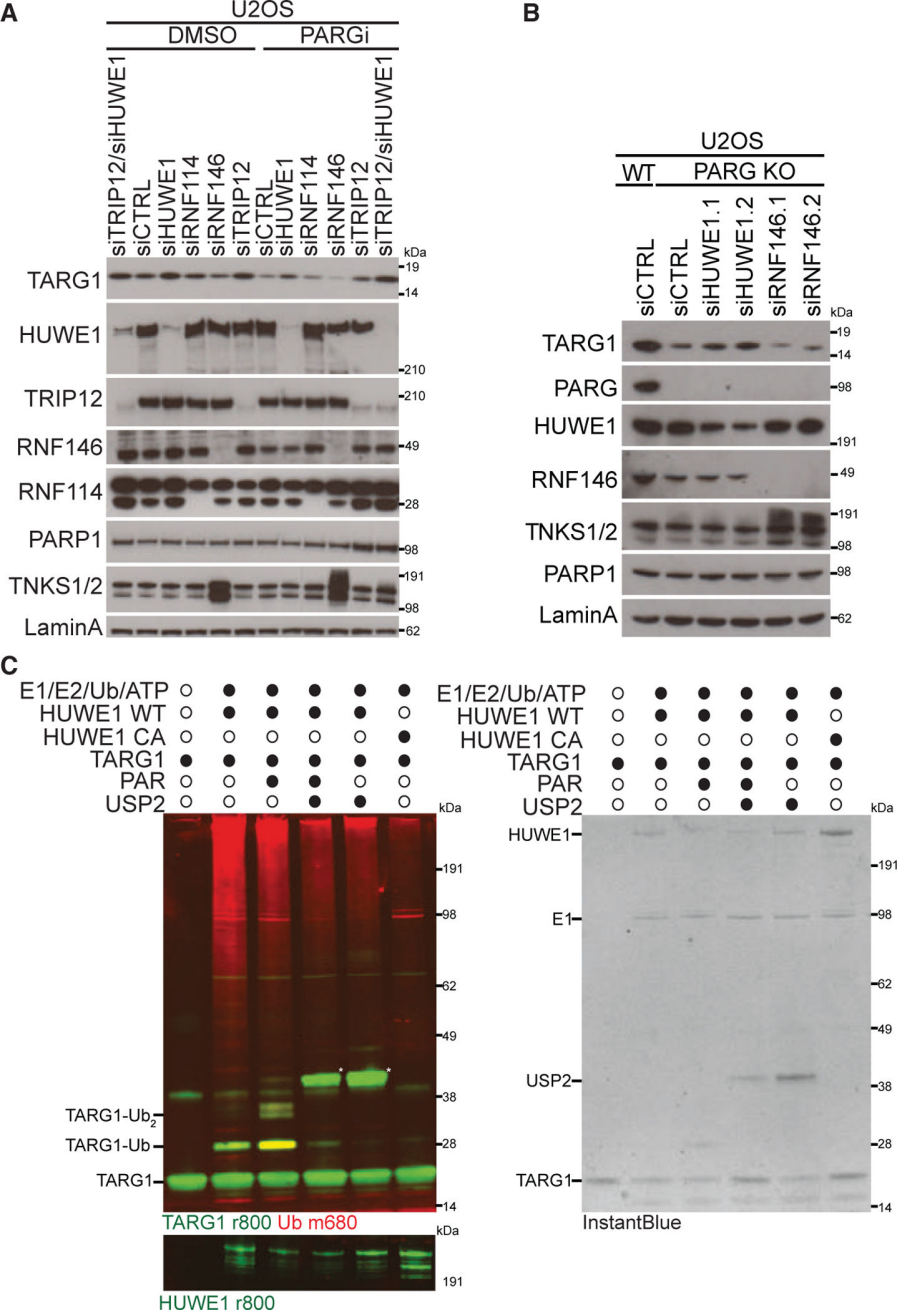
HUWE1 and TRIP12 target TARG1 for proteasomal degradation upon loss of PARG catalytic activity (A and B) U2OS cells were transfected with siRNAs as indicated and 24 h later treated with DMSO or 10 μM PARGi for 2 days (A). U2OS WT or PARG KO cells were lysed 48 h after siRNA transfection (B). TARG1 levels were analyzed by western blotting. siRNA transfection efficiency was checked using antibodies against RNF114, RNF146, TRIP12, and HUWE1. (C) Biochemical reconstitution of TARG1 ubiquitylation. TARG1-Ub was obtained by incubation of HUWE1 WT, E1, E2 (UbcH5B), ATP, and ubiquitin, in the absence or presence of an 18-mer PAR chain. The deubiquitylase USP2 cleaves the polyubiquitinated species. The HUWE1 CA mutant is inactive. The ubiquitylated products are visualized by western blotting using anti-mouse IRDye 680 and anti-rabbit IRDye 800 secondary antibodies. TARG1 and HUWE1 signals are shown in green, and the ubiquitin signal is shown in red. The non-specific signal resulting from cross-reactivity of the TARG1 antibody with USP2 is marked with *. (A–C) Three independent biological replicates were performed with similar results. See also Figure S3.

To uncover the missing link between PARylation and TARG1 degradation, we sought to identify the E3 ligases whose activities, stimulated by TNKS1/2 or PARP1/2-synthesized PAR chains, target TARG1 for proteasomal degradation. We transfected DMSO and PARGi-treated U2OS cells with small interfering RNAs (siRNAs) targeting four different E3 ligases—RNF146, HUWE1, RNF114, and TRIP12—and assessed their capacity to rescue TARG1 from PARGi-induced degradation (Figure 3A). RNF146, a well-established PAR-dependent ubiquitin ligase,^11,37,38^ was an obvious candidate to knock down. HUWE1 and TRIP12 were included as they both possess PAR-binding WWE domains,^10,17,39^ while RNF114 was selected as it has recently been characterized as a reader of ADPr and binder of a dual ADPr-ubiquitin hybrid modification that may be involved in protein degradation.^40–44^

Immunoblotting confirmed efficient knockdown of all four E3 ligases (Figure 3A). Consistent with the established findings, RNF146 knockdown stabilized TNKS1/2 protein levels, validating the role of the E3 ligase in promoting TNKS1/2 degradation^37^ (Figures 3A, 3B, and S3A). On the other hand, knockdown of RNF146 and RNF114 had no effect on TARG1 stability under basal or PARGi-treated conditions (Figure 3A). In contrast, depletion of HUWE1 or TRIP12 each partially stabilized TARG1 levels (Figures 3A and S3B), and their combined knockdown fully restored TARG1 levels, indicating that both E3 ligases contribute to the proteasomal degradation of TARG1 (Figure 3A). Moreover, HUWE1 knockdown also partially stabilized TARG1 in 293T cells (Figures S3A and S3C) and in U2OS-PARG KO cells (Figure 3B), demonstrating that HUWE1-mediated degradation of TARG1 occurs across cell types and in the context of PARG loss. Of note, HUWE1 depletion did not restore ALC1 levels (Figure S3C), indicating that the regulation of ALC1 is independent of HUWE1 activity.

While both HUWE1 and TRIP12 regulate TARG1 stability in cells, we focused our biochemical validation on HUWE1 to provide direct mechanistic evidence that one of the E3 ligases can ubiquitylate TARG1. To this end, we reconstituted an *in vitro* ubiquitylation reaction by incubating full-length HUWE1 with TARG1 and ubiquitin-processing components (E1, E2, ubiquitin, and ATP), and analyzed the reaction products by western blotting (Figure 3C). In the presence of all ubiquitylation components, an upward-shifted TARG1 band appeared, corresponding to mono-ubiquitylated TARG1 (Figure 3C, lane 2). Addition of an 18-mer PAR chain enhanced HUWE1 E3 ligase activity, as evidenced by increased mono-ubiquitylation and the appearance of an additional band corresponding to di-ubiquitylated TARG1 species (Figure 3C, lane 3). Our data thus indicate that the presence of PAR chains enhances ubiquitylation of TARG1. In contrast, incubation with the catalytically inactive HUWE1 C4341A (CA) mutant did not induce TARG1 ubiquitylation, confirming that the observed modifications are linked to the E3 ligase activity of HUWE1 (Figure 3C, lane 6). Further, treatment of the reaction mixture with the promiscuous deubiquitylase USP2 abolished the formation of the upward-shifted bands (Figure 3C, lanes 4–5), confirming that these species represented ubiquitylated forms of TARG1. Of note, the TARG1 antibody cross-reacted with recombinant USP2, resulting in a non-specific signal marked with an asterisk (Figure 3C). These findings thus validate our knockdown results by showing that HUWE1 can directly ubiquitylate TARG1. Collectively, these findings refine our model by identifying HUWE1 and TRIP12 as E3 ubiquitin ligases linking PARylation to TARG1 degradation.

## DISCUSSION

Although ADPr signaling plays essential roles in diverse cellular processes and is a key therapeutic anti-cancer target, the cellular functions and the regulatory mechanisms that underlie the activities of ADPr hydrolases remain poorly understood.^1,45^ While writers such as PARP1/2 and TNKS1/2 have been extensively studied, comparatively little is known about how their opposing erasers—PARG and MAR-hydrolases like TARG1—are regulated.

**Figure 4.**
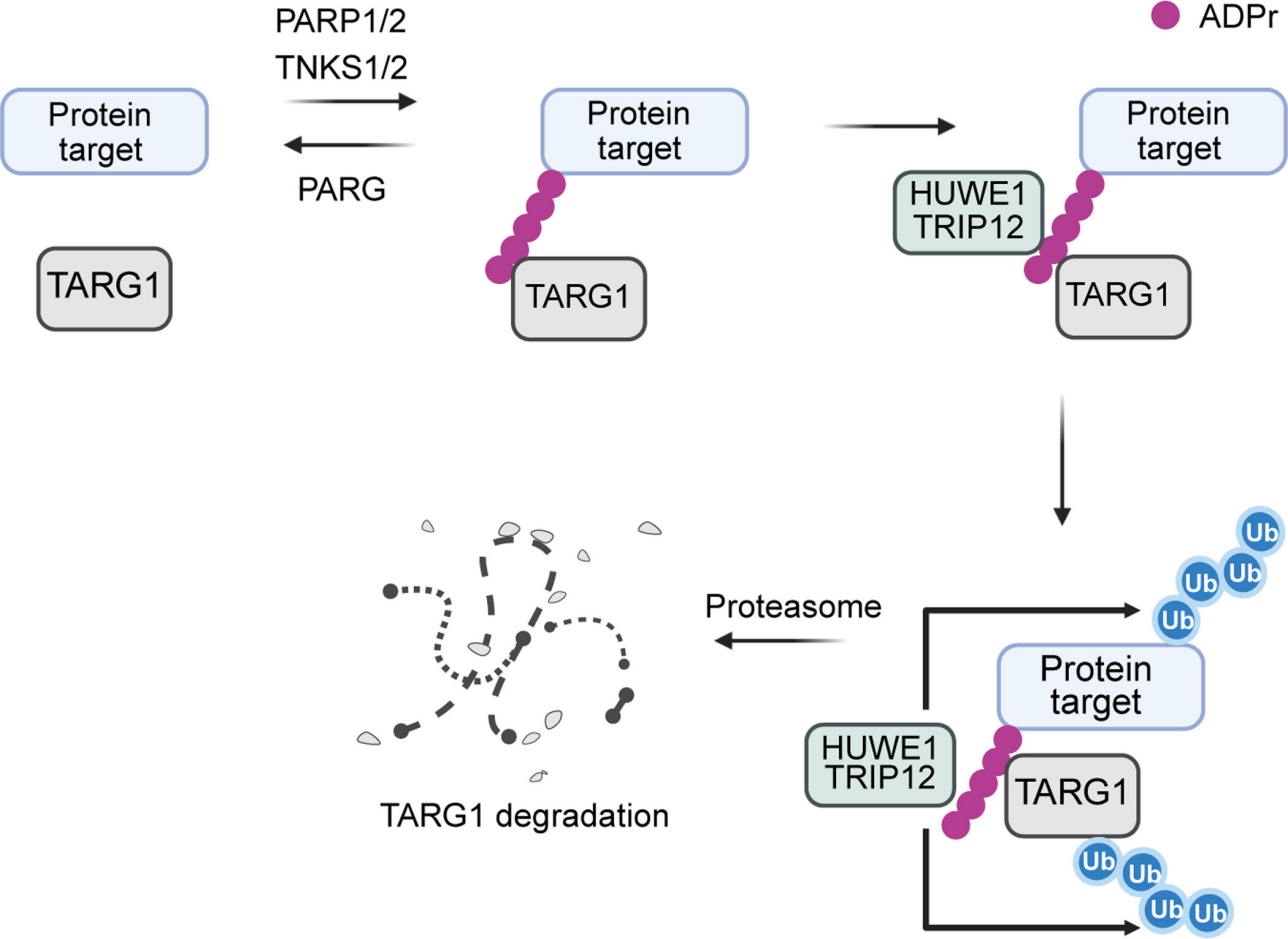
Proposed model for the PARG-mediated stabilization of TARG1 Schematic summarizing our current model for the molecular mechanism underlying TARG1 degradation: TARG1 may associate with PAR chains on automodified TNKS1/2, PARP1/2, or other PARylated substrate proteins, or it may itself be directly PARylated by TNKS1/2 or PARP1/2. These PAR chains, in turn, recruit the PAR-binding E3 ubiquitin ligases HUWE1 and TRIP12, leading to TARG1 ubiquitylation and proteasomal degradation. Under normal conditions, PARG removes these PAR chains, preventing HUWE1 and TRIP12 recruitment and thereby stabilizing TARG1. Upon PARG inhibition, persistent PAR chain accumulation promotes HUWE1 and TRIP12 recruitment to TARG1, enhancing its ubiquitylation and degradation.

In this study, we uncover a previously unrecognized role for PARG as a modulator of protein stability. Specifically, we show that PARG activity is required to prevent proteasomal degradation of TARG1, revealing an unanticipated layer of interplay between the two ADP-ribosylhydrolases. Mechanistic dissection of this regulation suggests that TARG1 associates with PAR chains on automodified PARP1/2, TNKS1/2, or other PARylated substrates, including TARG1 itself (Figure 4). These PAR chains may serve as molecular scaffolds for the recruitment of the PAR-binding E3 ubiquitin ligases HUWE1 and TRIP12, which in turn ubiquitylate TARG1 and target it for proteasomal degradation. Under normal conditions, PARG hydrolyses these PAR chains, thereby preventing the recruitment of HUWE1 and TRIP12 and maintaining TARG1 stability. Upon PARG inhibition, sustained PAR accumulation promotes HUWE1 and TRIP12 recruitment and consequent TARG1 degradation via the proteasome.

HUWE1, a WWE domain-containing E3 ligase, has previously been implicated in ADPr-dependent protein degradation pathways.^8^ Notably, a prior study suggested that PARP7/TiPARP recruits HUWE1 in an ADP-ribose-dependent manner to mediate the degradation of the transcription factor HIF-1α,^46^ pointing to a role for HUWE1 in ADPr-regulated ubiquitylation. Our findings reveal a clear parallel: we propose that HUWE1 may be similarly recruited to PAR chains synthesized by TNKS1/2 or PARP1/2 to promote TARG1 degradation (Figure 4). Together, these studies validate the hypothesis that HUWE1 functions as an ADPr-dependent E3 ligase, thereby joining other WWE domain-containing E3 ligases—such as RNF146 and TRIP12—capable of mediating ADPr-dependent ubiquitylation.^11,17,37,38^ Beyond TARG1 and HIF-1α, HUWE1 has been reported to regulate the degradation of many substrates, including histones and the DNA repair polymerase λ,^47–49^ which may also be controlled in an ADPr-dependent manner. The ADPr-dependent activity of TRIP12 has previously been established by showing its role in targeting auto-modified PARP1 for degradation.^17^ By identifying TARG1 as an additional substrate of TRIP12, our findings expand the repertoire of proteins regulated by TRIP12 through ADPr-dependent degradation.

As PARG and TARG1 cooperate to remove glutamate/aspartate-linked ADPr and PARylation,^25,26^ PARG may have evolved to control TARG1 levels to ensure sufficient TARG1 availability in order to maintain ADPr homeostasis. Moreover, in situations of excessive PARylation—such as during PARG inhibition—TARG1 could become sequestered on PAR chains or MAR, as it has previously been shown to form a covalent intermediate with ADPr.^26^ The degradation mechanism uncovered here could enable the removal of non-functional or stalled TARG1 molecules under these stress conditions (Figure 4).

Beyond TARG1, our quantitative proteomic analysis revealed that PARG inhibition affected the abundance of a small subset of other proteins, without corresponding transcriptional changes. Notably, the levels of the PAR-dependent chromatin remodeler ALC1 were reduced. While this suggests a role for PARG in modulating protein stability beyond TARG1, the extent to which these effects are direct—as demonstrated for TARG1—remains unclear. Moreover, the limited number of proteins found to be depleted upon PARGi treatment, which did not include any known PARdU targets, could be explained by functional redundancy among ADP-ribosylhydrolases, including ARH3, MACROD1/2, and others, which may compensate for the loss of PARG and thereby obscure the degradation of some substrates when PARG alone is inhibited.^21,24,28,50^ For instance, the joint hydrolytic activities of ARH3 and PARG have already been shown to stabilize PARP1 protein levels.^21^ Future studies combining the depletion of multiple hydrolases may help uncover additional proteins whose stability is maintained by PARG activity.

Our findings also carry important clinical implications. Indeed, PARG is emerging as a promising therapeutic target, especially in the context of PARPi resistance,^51^ and PARG inhibitors have now entered clinical trials.^52,53^ Our discovery that PARG inhibition destabilizes TARG1 raises important questions about how the loss of TARG1 might contribute to the cytotoxic effects of PARGi.^35,54^ Given TARG1’s role in genome maintenance, its degradation could either enhance therapeutic efficacy or introduce unwanted genomic instability—an area that merits further investigation. Beyond cancer, our data may also inform the studies of ADPr-hydrolases in neurodegenerative diseases, where deficiencies in enzymes such as TARG1, PARG, and ARH3 have been implicated.^26,55–57^

In addition, a recent study has linked PARG inhibition to enhanced degradation of proteolysis-targeting chimera (PRO-TAC) targets by showing that PARGi treatment increases the efficiency of PROTAC-mediated BRD2/3/4 degradation mediated by TRIP12.^58^ The ability of PARGi to promote protein degradation may have implications for combination therapies, as PROTACs and other targeted protein degradation strategies are emerging as powerful therapeutic tools.^59^ By enhancing PAR-dependent degradation, PARGi could potentially synergize with PROTACs, providing a rationale for future combination treatment strategies.

In summary, we identify a previously uncharacterized mechanism by which PARG safeguards TARG1 protein stability by pre-venting its PAR-dependent degradation mediated by HUWE1 and TRIP12. This work reveals an unrecognized function of PARG in regulating the stability of specific proteins and establishes TARG1 as a physiological substrate of PARdU. These findings offer insight into ADPr-dependent protein regulation, with implications for the development and optimization of PARG-targeted therapy, and further highlight the growing complexity of the ADPr-ubiquitin interplay,^60^ which is emerging as a key regulatory mechanism of physiological processes.

### Limitations of the study

At present, we cannot conclusively determine whether TARG1 is directly modified by PAR or whether it is instead recruited to PARylation hubs under stress conditions. Methodologically, profiling the modification status of TARG1 remains challenging, as ADPr homeostasis involves rapid and dynamic turnover driven by the coordinated actions of writers and erasers. In addition, TNKS1/2 have been proposed to generate ester-linked ADPr on glutamate and aspartate residues, producing modifications with high chemical lability that are difficult to detect. Compounding these challenges, PARylated TARG1 proteoforms are highly susceptible to degradation through PARdU pathways, further complicating their characterization.

Although we identify HUWE1 and TRIP12 as PARdU E3 ubiquitin ligases capable of degrading TARG1, additional redundant ligases may exist and remain uncharacterized. Future systematic investigation of this class of E3 ligases may reveal their individual substrate specificities and help distinguish redundant from non-redundant PARdU targets.

## RESOURCE AVAILABILITY

### Lead contact

Requests for further information, resources, and reagents should be directed to and will be fulfilled by the lead contact, Ivan Ahel (ivan.ahel@path.ox.ac.uk).

### Materials availability

All research reagents generated by the authors will be made available on request from the lead contact.

### Data and code availability

- The proteomics data have been deposited to the ProteomeXchange Consortium via the partner repository,^61^ with the dataset identifier PRIDE: PXD065407. The genomics data have been deposited in NCBI’s Gene Expression Omnibus and are accessible through the GEO series accession number GEO: GSE310746.
- This paper did not generate original code.
- Any additional information required to reanalyze the data reported in this paper is available from the lead contact upon request.

## STAR★METHODS

### EXPERIMENTAL MODEL AND STUDY PARTICIPANT DETAILS

#### Cell culture

Human female osteosarcoma U2OS (ATCC HTB-96), human embryonic kidney (HEK) 293T (ATCC CRL-3216) and human female cervix adenocarcinoma HeLa (ATCC CLL-2) cells were acquired from ATCC and grown in DMEM (Sigma) supplemented with 10% FBS (Gibco) and penicillin-streptomycin (100 U/mL, Gibco). Kuramochi (CVCL_1345, female) cells were cultured in RPMI-1640 (Sigma) supplemented with 20% FBS (Gibco) and penicillin-streptomycin (100 U/mL, Gibco). Authenticated WT cell lines were obtained from ATCC or JCBR cell bank. Knockout lines were validated by western blotting. Cell lines were screened regularly to ensure they tested negative for mycoplasma.

#### Generation of cell lines

The U2OS TARG1 knock-out cell lines were generated as described previously.^63^ They were generated by CRISPR/Cas9 following the published protocol.^66^ The following gDNA sequences were targeted: CACCGAGGATTGTCGCATGGGCGCT; AAACAGCG CCCATGCGACAATCCTC. Annealed primers were cloned into pSpCas9(BB)-2A-GFP (PX458) and after sequencing verification, the plasmid was transfected into U2OS cells. 1–2 days post-transfection, single GFP-positive cells were sorted with a FACSAria II into 96-well plates. Monoclonal cell lines were tested for TARG1 deficiency by anti-TARG1 Western blot. pSpCas9(BB)-2A-GFP (PX458) was a gift from Feng Zhang (Addgene plasmid #48138).

The U2OS PARG KO cells were generated by CRISPR/Cas9. The following gDNA sequences were targeted: ACCAGTTGGA TGGACACTAAAGG; GCAGACTACAGAAGATGAACAGG; GAGACGCTGACATTGAATTTAGG; TGAGAAGAATGCCTCGGTGTGGG.

LentiCRISPR v2 plasmids (Addgene #52961) containing PARG sgRNAs were transfected with Lipofectamine 2000 (Life Technologies) into 293T cells along with psPAX2 and pMD.G packing plasmids to produce lentivirus. Media was changed 16 h after transfection, and the virus was harvested and filtered ~48 h after the media was changed and stored at - 80°C or used to infect U2OS for 2 days. Then, cells were selected with 2 μg/mL puromycin for 1 week before performing single-cell sorting in 96-well plates to isolate individual clones. Clones were tested by Western blot for the absence of PARG protein expression.

U2OS TARG1 KO cells complemented with TARG1-WT or catalytically inactive K84A were generated by lentivirus transduction as described previously.^63^

### METHOD DETAILS

#### Western blotting

Cells were lysed with Triton X-100 lysis buffer (50 mM Tris-HCl pH 8.0, 100 mM NaCl, 1% Triton X-100) supplemented with 2.5 mM MgCl_2_, protease and phosphatase inhibitors (Roche), Olaparib (Cayman Chemical; 1 μM for U2OS and 2 μM for 293T cells), PARGi PDD00017273 (Sigma; 1 μM) and TNKSi XAV939 (Sigma; 1 μM) at 4°C. The lysates were incubated with 0.05% Benzonase (Sigma) for 30 min at 4°C. Protein concentrations were analyzed by Bradford Protein Assay (BioRad). Proteins were boiled for 3min in 1x NuPAGE LDS sample buffer (Invitrogen) with 40 mM DTT, resolved on NuPAGE Novex 4–12% Bis-Tris gels (Invitrogen) or NuPAGE 3–8% Tris-Acetate gels (Invitrogen) when examining HUWE1 or TRIP12 levels, and transferred onto nitrocellulose membranes (BioRad) using Trans-Blot Turbo Transfer System (BioRad). The membranes were blocked in PBS buffer with 0.1% Tween 20 and 5% non-fat dried milk for 1H at room temperature and incubated overnight with primary antibodies (1:1000, unless stated otherwise) at 4°C, followed by 1-h incubation with peroxidase-conjugated secondary anti-mouse (Agilent, P0447, 1:3000) or anti-rabbit (Agilent, P0399, 1:3000) antibody at room temperature.

Rabbit anti-TARG1 (25249–1-AP, 1:500) antibody was from Proteintech. Rabbit anti-histone H3 (07–690, 1:5000) antibody was from Millipore. Rabbit anti-PARP1 (ab32138; 1:2000), anti-Lamin-A (ab26300), anti-GFP (ab290, 1:3000) and mouse anti-ALC1 (ab51324) antibodies were from Abcam. Rabbit anti-HUWE1/Lasu1/Ureb1 (A700–129, 1:500) and Rabbit anti-TRIP12 (A301–814A) antibodies were from Bethyl. Mouse anti-RNF146 (H00081847-B01P, 1:500) and anti-PARG (H00008505-B01P, 1:500) antibodies were from Abnova. Mouse anti-Tankyrase1/2 (sc-365897, 1:500) antibody was from Santa Cruz. Mouse anti-PARP2 (ALX-804–639-L001, 1:500) antibody was from Enzo Life Sciences. Rabbit anti-PARP3 (NBP1–31415, 1:500) antibody was from Novus. Rabbit anti-poly/mono-ADP Ribose (89190S) and anti-PARG (66564, 1:500) were from Cell Signaling. Rabbit anti-RNF114 (hpa021184) was from Atlas Antibodies. Blots were developed using ECL (Invitrogen) and analyzed by exposing them to films.

#### siRNA transfection

siRNA transfection was performed using Lipofectamine RNAiMAX (Invitrogen) and 20 nM siRNA according to the manufacturer’s instructions. Silencer Select Negative Control No. 1 siRNA, HUWE1.1 (s19596), HUWE1.2 (s19597), HUWE1.3 (s19595), RNF146.1 (s37822), RNF146.2 (s37823), TRIP12 (s17810) and RNF114 (s31751) were purchased from Ambion (Invitrogen).

#### Immunoprecipitation

293T cells were plated, cultured overnight and transfected using Polyfect (Qiagen) with YFP-tagged TARG1 WT, TARG1 mutant or empty vector for 24H. Cells lysates were obtained as described for western blotting. Protein concentrations were normalised, after which the cell lysates were incubated with GFP-Trap magnetic agarose beads (ChromoTek) for 2 h whilst rotating at 4°C. The beads were pelleted using a magnetic separator rack and washed five times with Triton X-100 lysis buffer, eluted with 2x NuPAGE LDS sample buffer (Invitrogen) with DTT (Sigma-Aldrich), boiled for 5 min at 95°C, and analyzed by Western blotting.

#### Cloning, protein expression and purification

Human HUWE1 was codon optimized for insect cell expression and synthesized in fragments before assembly into a GoldenBac pGBdest vector using a BsaI-GoldenGate reaction. The construct had an N-terminal his_8_ tag followed by a rigid enhancer linker (AEAAAKEAAAKEAAAKEAAAKALEAEAAAKEAAAKEAAAKEAAAKA) and a TEV cleavage site before the HUWE1 open reading frame. The C4341A mutation was generated by introduction into the corresponding fragment before GoldenGate assembly. Plasmids were transformed into DH10 MultiBac cells to generate bacmids. *Spodoptera frugiperda* (Sf9) cells were grown in ESF921 serum-free growth medium (Expression Systems) and transfected with the bacmids for amplification of the baculovirus and this was monitored using yellow fluorescent protein signal. HUWE1 expression was performed in in *Trichoplusia ni* High-Five insect cells (Thermo Fisher) at a density of 1.5 × 10^6^ using a 1:70 inoculation from the V1 stock. After 92 h at 21°C in Insect Xpress Protein-free Insect Cell Medium (Lonza) supplemented with GlutaMAX (GIBCO) and Pen/Strep Amphotericin B (Lonza), cells were harvested by centrifugation at 700 x g, washed in PBS and then flash frozen and stored at −70°C.

For purification, the thawed pellet was resuspended in 50 mM HEPES pH 7.5, 300 mM NaCl, 0.5 mM TCEP, 20 mM imidazole with Complete EDTA-free Protease inhibitor (Roche) and 20 μL Benzonase (IMP Molecular Biology Service) and lysed by douncing. Centrifugation at 40,000 x g was used to clear the lysis before loading on a 5 mL HisTrap HP column (Cytiva) equilibrated in the lysis buffer using an Äkta Pure 25 system (Cytiva). The column was washed with 10 column volumes of the same buffer, followed by 7 column volumes of the same buffer but with 35 mM imidazole, and then elution using 300 mM imidazole. The protein was cleaved overnight with TEV protease and simultaneously dialyzed to remove imidazole. The cleaved protein was then reapplied to the HisTrap HP column in 20 mM imidazole and the flow through was collected and concentrated to 1.5 mL. Finally, the protein was applied to a Superose 6 16/60 column (Cytiva) equilibrated in 50 mM HEPES pH 7.5, 150 mM NaCl, 0.5 mM TCEP. Protein-containing fractions were pooled and concentrated. The protein was pure as assessed by SDS-PAGE and the concentration was estimated by A280 absorption using an extinction coefficient of 251,770 M^−1^ cm^−1^.

WT and TARG1 K84A recombinant proteins were expressed and purified as previously described.^26^ His-E1, UbC5Hb and USP2 were generated as previously described.^42^ TARG1 cloning was performed by Gateway cloning (Invitrogen) according to the manufacturer’s instructions. Using Gateway LR Clonase II (Invitrogen), pDONOR221-TARG1 vector was cloned into pDEST-N-YFP/FRT/TO pcDNA5. To obtain the TARG1 K84A construct, the point mutation was produced using QuikChange Lightning site-directed mutagenesis kit (Agilent) according to the manufacturer’s instructions.

#### *In vitro* ubiquitylation assays

Reactions were performed in a total volume of 18 μL with 0.2 μM E1, 0.5 μM UbcH5b, 10mM Mg-ATP, 50 μM ubiquitin in 50mM HEPES Na pH 7.5 and 50 mM NaCl. Reactions were pre-incubated for 30 min to allow E2 charging, then initiated by the addition of 0.1 μM wild-type or mutant HUWE1, 2 μM TARG1, and 2 μM 18-mer PAR chain in the indicated conditions, followed by incubation for 30 min at room temperature. 18-mer PAR chain was synthesized enzymatically as previously described.^19,67^

For USP2 treatment, ubiquitylated TARG1 species were generated as described above and subsequently incubated with 1 μM USP2 for a further 30 min at 37°C. Reactions were stopped by adding 3X NuPAGE LDS (Invitrogen) and 100mM DTT. Samples were loaded onto SDS PAGE (4–12% Bis-Tris gel/MOPS buffer) and visualised by Instant Blue stain or transferred onto nitrocellulose membrane (BioRad TransBlot Turbo), and blocked with 5% BSA for 1H at room temperature. The membrane was incubated with primary antibodies (rabbit polyclonal anti-TARG1, Proteintech 25249–1-AP, or mouse anti-ubiquitinylated protein FK2, 04–263 Millipore, or rabbit HUWE1/Lasu1/Ureb1 Bethyl A700–129) overnight at 4°C followed by secondary antibodies incubation (goat anti-rabbit IgG secondary 800CW, 926–32211, Li-COR Biosciences; or goat anti-mouse IgG secondary 680RD, 926–68070, Li-COR Biosciences) for 1 h at room temperature. Membranes were washed in in 0.1% PBS-Tween 20 (PBST) and scanned using Li-Cor Odyssey CLx image (LI-COR Biosciences).

#### Proteomics data generation

HeLa and U2OS cells were grown in 15-cm dishes in quadruplicate, and treated with DMSO or 25 μM PARGi for 4 days. Cells were washed twice with ice-cold PBS, and afterward scraped in 3 mL of ice-cold phosphate-buffered saline (PBS), transferred to 15 mL tubes, and pelleted by centrifugation at 500*g* for 2 min. PBS was decanted, and cell pellets were vigorously lysed in 3 mL of 6 M guanidine-HCl, 50 mM TRIS pH 8.5. Lysates were homogenized through sonication at 30 W for two pulses of five seconds. A standard Bradford protein assay was used to determine concentration of proteins in lysates, and 200 μg of total protein from each sample was subsequently digested using 1:80 (w/w) endoproteinase Lys-C (Wako), for 4.5 h at room temperature. Digests were diluted using 3 volumes of ice-cold 50 mM TRIS pH 8.5, and afterward digested overnight with 1:66 (w/w) sequencing-grade trypsin (Promega).

Digests were fractionated at high pH, on-StageTip. To this end, samples were first acidified by addition of trifluoroacetic acid until a final concentration of 1% (v/v), and clarified through 0.45 μm spin filters at 12,000*g*. Supernatants were transferred to clean tubes, and basified by addition of ammonium hydroxide to a final concentration of 0.5% (v/v). Quadlayer StageTips were prepared using four punch-outs of C18 material (Sigma-Aldrich, Empore SPE Disks, C18, 47 mm). StageTips were equilibrated using 100 μL of methanol, 100 μL of 80% acetonitrile (ACN) in 200 mM ammonium hydroxide, and two times 100 μL 50 mM ammonium hydroxide. Samples were loaded, and StageTips were subsequently washed twice with 100 μL 50 mM ammonium hydroxide, and afterward eluted as eight HpH fractions (F1–8) using 80 μL of 2%, 4%, 6%, 8%, 11%, 14%, 18%, and 25% ACN in 50 mM ammonium hydroxide. All fractions were dried to completion in LoBind tubes, using a SpeedVac for 3 h at 60°C, after which the dried peptides were dissolved using 11 μL of 0.1% formic acid. Approximately 25% of all fractions, corresponding to ~5 μg of digested protein, were analyzed using LC-MS.

#### Mass spectrometry data acquisition

All MS samples were analyzed on an EASY-nLC 1200 system (Thermo) coupled to an Orbitrap Exploris 480 mass spectrometer (Thermo). Separation of peptides was performed using 20-cm columns (75 μm internal diameter) packed in-house with ReproSilPur 120 C18-AQ 1.9 μm beads (Dr. Maisch). Elution of peptides from the column was achieved using a gradient ranging from buffer A (0.1% formic acid) to buffer B (80% acetonitrile in 0.1% formic acid), at a flow of 250 nL/min. The total gradient length was 90 min per sample, including ramp-up and wash-out, with an analytical gradient of 70 min which was customized for each fraction. To this end, fraction 1 was analyzed with a gradient ranging from 5% B to 30% B, and each subsequent fraction had a stepwise increase in buffer B% with the final fraction 8 ranging from 13% B to 41% B. Analytical columns were heated to 40°C using a column oven, and ionization was achieved using a NanoSpray Flex NG ion source. Spray voltage set to 2 kV, ion transfer tube temperature to 275°C, and RF funnel level to 40%. The full scan range was set to 300–1300 m/z, MS1 resolution to 120,000, MS1 AGC target to ‘‘200’’ (2,000,000 charges), and MS1 maximum injection time to ‘‘Auto’’. Precursors with charges 2–6 were selected for fragmentation using an isolation width of 1.3 m/z, and fragmented using higher-energy collision disassociation (HCD) with normalized collision energy of 25. Precursors were excluded from re-sequencing by setting a dynamic exclusion of 70 s. MS2 AGC target was set to ‘‘200’’ (200,000 charges), and MS2 maximum injection time to ‘‘Auto’’. The MS2 resolution was set to 15,000, intensity threshold to 430,000 charges per second, and number of dependent scans (TopN) to 18.

### QUANTIFICATION AND STATISTICAL ANALYSIS

#### RNA sequencing data generation and analysis

U2OS cells were treated with DMSO or 25 μM PARGi for 4 days. RNA extraction, subsequent sequencing and analysis was performed as previously described,^21^ with the aligned reads aggregated on the Gencode V41 annotation with HTseq-count version 0.11.3.^68^ Read counts were imported into R version 4.0.5. and differential expression analysis was performed using the *DESeq2* and *apeglm* packages. Genes were selected as either up-regulated or down-regulated with a log2FoldChange of > |1| and adjusted *p*-value <0.05.

#### MS data processing

All RAW files were analyzed using MaxQuant software version 1.5.3.30 using default settings,^64^ except with fast label-free quantification enabled with 3+ peptide pairs using the maxLFQ algorithm.^69^ We also allowed matching between runs (alignment time of 1 min within a 20 min window), and allowed up to 3 missing cleavages per peptide. The human FASTA database used in this study was downloaded from Uniprot on the 25th of June, 2023 (UP000005640). All data was filtered by posterior error probability to achieve a false discovery rate of <1% at the PSM- and protein level (default), at both the peptide-spectrum match and the protein assignment levels.

#### MS data filtering and statistical analysis

The protein groups matrix was further processed using a combination of the freely available Perseus software,^65^ and the statistical programming language R (version 4.5 ‘‘How About a Twenty-Six’’). This filtering includes log2 transformations, *n* = 4 filtering within at least one group, matrix-wise imputation (down shift 1.8, width 0.30), two-sample t-tests for differential expression (at 5% permutation-based FDR with S0 = 0.1). We also filtered potential contaminants, reverse hits, and peptides which were never identified by their unmodified peptides. Prior to imputation and statistical testing, we split the matrix into U2OS and HeLa specific protein identifications, to prevent imputation of proteins which were never detected in any sample for that cell line (but may have been reliably detected in the other).

## Supplementary Material

1

Data S1

SUPPLEMENTAL INFORMATION

Supplemental information can be found online at 10.1016/j.celrep.2025.116789.

## Figures and Tables

**Table:** KEY RESOURCES TABLE

REAGENT or RESOURCE	SOURCE	IDENTIFIER
Antibodies		

TARG1 (rabbit polyclonal)	Proteintech	Cat# 25249–1-AP; RRID: AB_2753118
histone H3 (rabbit polyclonal)	Millipore	Cat# 07–690; RRID: AB_417398
Poly/mono-ADP-ribose (rabbit monoclonal)	Cell Signaling	Cat# 89190S; RRID: AB_3716623
PARG (rabbit monoclonal)	Cell Signaling	Cat# 66564; RRID: AB_2750890
PARG (mouse polyclonal)	Abnova	Cat# H00008505-B01P; RRID: AB_1677774
RNF146 (mouse polyclonal)	Abnova	Cat# H00081847-B01P; RRID: AB_1580189
PARP2 (mouse monoclonal)	Enzo Life Sciences	Cat# ALX-804–639-L001; RRID: AB_2052178
PARP3 (rabbit polyclonal)	Novus	Cat# NBP1–31415; RRID: AB_2299147
PARP1 (rabbit monoclonal)	Abcam	Cat# ab32138; RRID: AB_777101
LaminA (rabbit polyclonal)	Abcam	Cat# ab290; RRID: AB_303395
ALC1 (mouse monoclonal)	Abcam	Cat# ab5132; RRID:AB_869125
HUWE1 (rabbit monoclonal)	Bethyl	Cat# A700–129; RRID: AB_289192
TRIP12 (rabbit polyclonal)	Bethyl	Cat# A301–814A; RRID: AB_1264344
RNF114 (rabbit polyclonal)	Atlas Antibodies	Cat# HPA021184 RRID: AB_1859185
TNKS1/2 (mouse monoclonal)	Santa Cruz	Cat# sc-365897 RRID: AB_10844977
Ubiquitinylated protein FK2 (mouse monoclonal)	Millipore	Cat#04–263 RRID: AB_612093
GFP (rabbit polyclonal)	Abcam	Cat# ab290; RRID: AB_303395
Goat polyclonal anti-mouse, HRP-conjugated	Agilent	Cat# P0447; RRID: AB_2617137
Swine polyclonal anti-rabbit, HRP-conjugated	Agilent	Cat# P0399; RRID: AB_2617141
IRDye® 680RD goat anti-mouse	Li-Cor	Cat# 926–68070; RRID: AB_10956588
IRDye® 800CW goat anti-rabbit	Li-Cor	Cat# 926–32211; RRID: AB_2651127

Chemicals and recombinant proteins		

PDD00017273	Sigma	Cat# SML1781
Olaparib	Cayman Chemical	Cat# 10621
XAV939	Sigma	Cat# X3004
MG-132	Sigma	Cat# 474787
MLN4924	Cayman Chemical	Cat# 15217
ATP	Thermo Fisher	Cat# R0441

Ubiquitin	R&D Systems	Cat# U-100H-10M
Recombinant TARG1 WT protein	Sharifi et al.^26^	N/A
Recombinant TARG1 K84A protein	Sharifi et al.^26^	N/A
Recombinant human HUWE1 WT protein	This paper	N/A
Recombinant human HUWE1 C4341A protein	This paper	N/A
Recombinant USP2 protein	Zhu et al.^62^	N/A
cOmplete™, EDTA-free Protease Inhibitor Cocktail	Sigma	Cat# 11873580001
PhosSTOP	Sigma	Cat# 4906845001
Benzonase	Sigma	Cat# 1016970001
NuPAGE LDS sample buffer	Invitrogen	Cat# NP0007
NuPAGE Novex 4–12% Bis-Tris gel	Invitrogen	Cat# WG1402A
NuPAGE 3–8% Tris-Acetate gels	Invitrogen	Cat# WG1602A

Critical commercial assays		

Lipofectamine RNAiMAX	Invitrogen	Cat# 3778075
QuikChange Lightning Site-Directed Mutagenesis Kit	Agilent	Cat# 210519
LR Clonase II enzyme mix	Invitrogen	Cat# 11791020
Polyfect Transfection Reagent	Qiagen	Cat# 301105
GFP-Trap Magnetic Agarose	Chromotek	Cat# gtma-20

Deposited data		

Raw MS data	This paper	PRIDE: PXD065407
RNA-seq data	This paper	GEO: GSE310746

Experimental models: Cell lines		

Human: U2OS cells	ATCC	Cat# HTB-96
Human: HEK 293T cells	ATCC	Cat# CRL-3216
Human: U2OS TARG1 KO cells	Tromans-Coia et al.^63^	N/A
Human: U2OS TARG1 KO cells complemented with untagged TARG1 WT	Tromans-Coia et al.^63^	N/A
Human: U2OS TARG1 KO cells complemented with untagged TARG1 K84A	Tromans-Coia et al.^63^	N/A
Human: Kuramochi cells	JCRB Cell Bank	Cat# JCRB0098
Human: HeLa cells	ATCC	Cat# CLL-2
Human: U2OS PARG KO cells	This paper	N/A
Insect: *Trichoplusia ni* High-Five	ThermoFisher Scientific	Cat# B85502<

Oligonucleotides		

sgRNA targeting PARG exon 1 ACCAGTTGGATGGACACTAAAGG	IDT	N/A
sgRNA targeting PARG exon 2 (2/5) GCAGACTACAGAAGATGAACAGG	IDT	N/A
sgRNA targeting PARG exon 2 (4/5) GAGACGCTGACATTGAATTTAGG	IDT	N/A
sgRNA targeting PARG exon 3 TGAGAAGAATGCCTCGGTGTGGG	IDT	N/A
sgRNA targeting TARG1 exon 3 GGATTGTCGCATGGGCGCT	IDT	N/A
sgRNA targeting TARG1 intron 3 GGTAAACGTCTAAACTAG	IDT	N/A
Silencer™ Select HUWE1.1 siRNA	Invitrogen	Cat# s19596
Silencer™ Select HUWE1.2 siRNA	Invitrogen	Cat# s19597
Silencer™ Select HUWE1.3 siRNA	Invitrogen	Cat# s19595
Silencer™ Select Negative Control No. 1 siRNA	Invitrogen	Cat# 4390843
Silencer™ Select RNF146.1 siRNA	Invitrogen	Cat# s37822
Silencer™ Select RNF146.2 siRNA	Invitrogen	Cat# s37823

Silencer™ Select RNF114 siRNA	Invitrogen	Cat# s31751
Silencer™ Select TRIP12 siRNA	Invitrogen	Cat# s17810

Recombinant DNA		

pDONR221<	Invitrogen	Cat# 12536017<
pDEST12.2<	Invitrogen	Cat# 11808–011<
pDEST-YFP-TARG1 WT	This paper	N/A
pDEST-YFP-TARG1 K84A	This paper	N/A
pGBdest-HUWE1 WT	This paper	N/A
pGBdest-HUWE1 C4341A	This paper	N/A

Software and algorithms		

MaxQuant 1.5.3.30	Cox and Mann^64^	N/A
Perseus	Tyanova et al.^65^	N/A
Prism 7	GraphPad	N/A

## Highlights

- PARG inhibition leads to the depletion of TARG1 and a subset of other proteins
- PARG inhibitor-induced depletion of TARG1 is proteosome and PARylation dependent
- The E3 ligases HUWE1 and TRIP12 target TARG1 for proteasomal degradation
