# Supplementary material for: PARG regulates the proteasomal degradation of TARG1
Source: Cell Rep. Author manuscript; Available in PMC 2026 Jul 4. (PMC13332792; doi:10.1016/j.celrep.2025.116789)
Supplement: 1 [file NIHMS2142882-supplement-1.pdf]

Cell Reports, Volume *45*

Supplemental information

PARG regulates the proteasomal degradation of TARG1

Joséphine Groslambert, Sara C. Buch-Larsen, Ivo A. Hendriks, Robert Kurzbauer, Jonas D. Elsborg, Chatrin Chatrin, Thomas Agnew, Callum Henfrey, Michael Tellier, Evgeniia Prokhorova, Song My Hoang, Jonathan Barosso-Gonzalez, Roderick J. O'Sullivan, Tim Clausen, Michael L. Nielsen, and Ivan Ahel

Figure S1. The loss of PARG catalytic activity leads to the depletion of TARG1 protein levels, related to Figure 1.

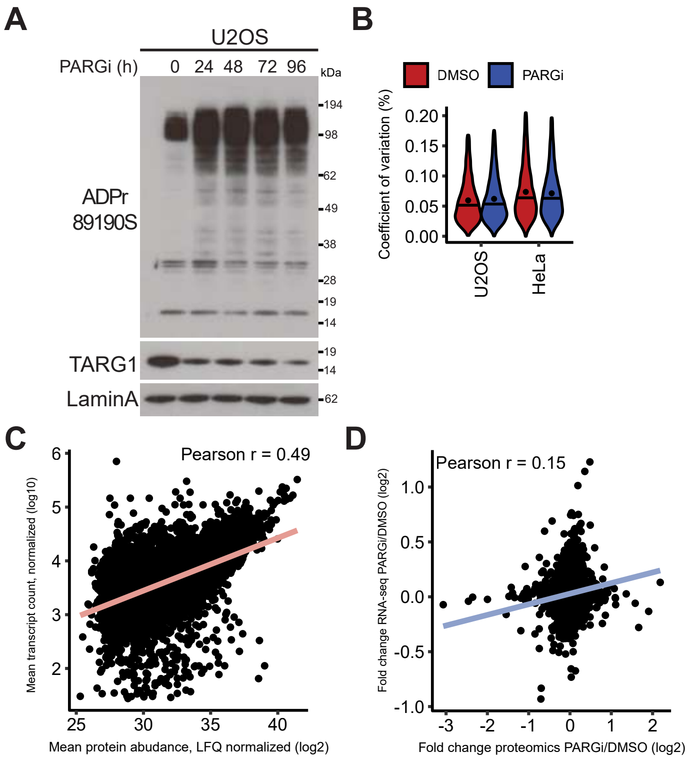

Figure S2. PARG regulates the proteasomal degradation of TARG1 in a PARylation dependent-manner, related to Figure 2.

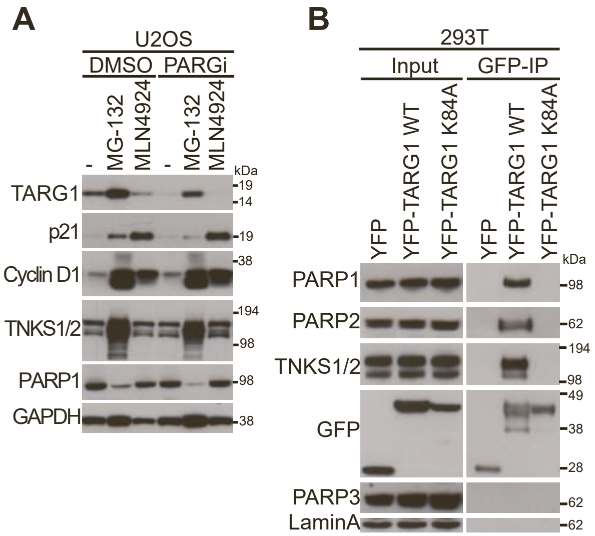

Figure S3. HUWE1 targets TARG1 for proteasomal degradation upon PARG inhibition, related to Figure 3.

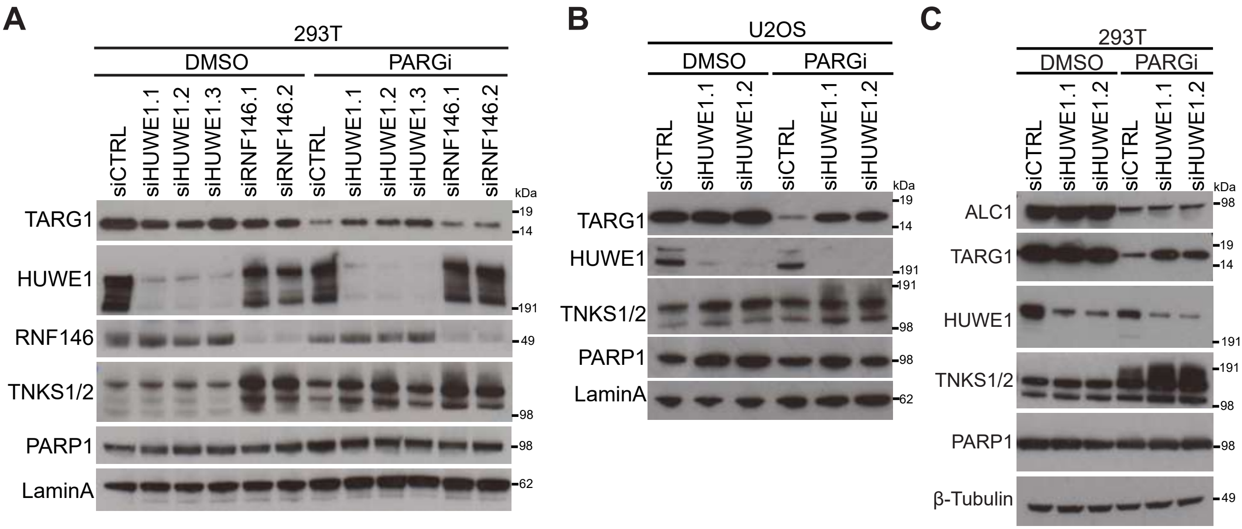

Supplemental Figure Legends

Figure S1. The loss of PARG catalytic activity leads to the depletion of TARG1 protein levels, related to Figure 1.

(**A**) U2OS cells were treated with 10 µM PARGi as indicated. ADPr and TARG1 levels were analysed by western blotting.
(**B**) Violin plots depicting the distribution of the coefficient of variation (CV) within the different experimental conditions. The flat line corresponds to the median CV, while the dots mark the mean CV of the distributions.
(**C**) Pearson correlation coefficient (r) between global transcriptome and proteome expression levels in U2OS cells.
(**D**) Pearson correlation coefficient (r) between the $\log_2$ fold changes in the transcriptome and proteome upon PARGi-treatment of U2OS cells.

Figure S2. PARG regulates the proteasomal degradation of TARG1 in a PARylation dependent-manner, related to Figure 2.

(**A**) U2OS cells were treated with DMSO or 10 µM PARGi for 3 days. 2.5 µM MG-132 or 1 µM MLN4934 was added for the last 24 h of PARGi treatment. TARG1 levels were analysed by western blotting.
(**B**) 293T cells were transfected with the indicated plasmids 24H prior lysis. Cells lysates and GFP-immunoprecipitations (GFP-IPs) were analysed by western blotting with the indicated antibodies.
At least two independent biological replicates were performed with similar results.

Figure S3. HUWE1 targets TARG1 for proteasomal degradation upon PARG inhibition, related to Figure 3.

(A-C) 293T (A and C**)** and U2OS (B) cells were transfected with siRNAs as indicated and 24h later treated with DMSO or 10 µM PARGi for 2 days. TARG1 levels were analysed by western blotting. siRNA transfection efficiency was checked using antibodies against RNF146 and HUWE1.
Three independent biological replicates were performed with similar results.
